# On the Construction of Group Equivariant Non-Expansive Operators *via* Permutants and Symmetric Functions

**DOI:** 10.3389/frai.2022.786091

**Published:** 2022-02-15

**Authors:** Francesco Conti, Patrizio Frosini, Nicola Quercioli

**Affiliations:** ^1^Department of Mathematics, University of Pisa, Pisa, Italy; ^2^Institute of Information Science and Technologies “A. Faedo”, National Research Council of Italy (CNR), Pisa, Italy; ^3^Department of Mathematics, University of Bologna, Bologna, Italy; ^4^Alma Mater Research Center on Applied Mathematics, University of Bologna, Bologna, Italy; ^5^Alma Mater Research Institute for Human-Centered Artificial Intelligence, University of Bologna, Bologna, Italy; ^6^Research Centre on Electronic Systems for the Information and Communication Technology, University of Bologna, Bologna, Italy; ^7^ENEA Centro Ricerche Bologna, Bologna, Italy

**Keywords:** GENEO, permutant, symmetric function, persistence diagram, persistent homology, machine learning

## Abstract

Group Equivariant Operators (GEOs) are a fundamental tool in the research on neural networks, since they make available a new kind of geometric knowledge engineering for deep learning, which can exploit symmetries in artificial intelligence and reduce the number of parameters required in the learning process. In this paper we introduce a new method to build non-linear GEOs and non-linear Group Equivariant Non-Expansive Operators (GENEOs), based on the concepts of symmetric function and permutant. This method is particularly interesting because of the good theoretical properties of GENEOs and the ease of use of permutants to build equivariant operators, compared to the direct use of the equivariance groups we are interested in. In our paper, we prove that the technique we propose works for any symmetric function, and benefits from the approximability of continuous symmetric functions by symmetric polynomials. A possible use in Topological Data Analysis of the GENEOs obtained by this new method is illustrated.

## 1. Introduction

In recent years, the theory of equivariant operators has become a topic of great interest to the scientific community, since these operators allow to make explicit the use of symmetries in deep learning and artificial intelligence (Mallat, 2012, 2016; Bengio et al., 2013; Zhang et al., 2015; Anselmi et al., 2016, 2019; Cohen and Welling, 2016; Worrall et al., 2017), thereby reducing the number of parameters required in the learning process. In particular, group equivariant non-expansive operators (GENEOs) have been recently proposed as elementary components for building new kinds of neural networks, benefiting from good mathematical properties, such as compactness and convexity, under suitable assumptions on the space of data and with respect to the choice of appropriate topologies (Bergomi et al., 2019). In particular, compactness guarantees total-boundedness, i.e., for every ε > 0 we can find a finite set $F=\left\{F_{1},\ldots ,F_{s}\right\}$ of GENEOs such that any other GENEO has a distance less than ε from at least one *F*_*i*_. This property opens the way to the search for methods to effectively build such a representative set F, leading us to look for new techniques to produce GENEOs.

GENEOs are grounded in Topological Data Analysis (TDA) and allow to shift the attention from the data to the observers who process them, and to the properties of invariance and simplification associated with those observers. The use of these operators is justified by the fact that in most of the cases we are not directly interested in data, but in approximating the experts' behavior in the presence of given data (Frosini, 2016). Since different agents can have different reactions in the presence of the same data, it is clear that data analysis has to be based on each pair (data, observer) rather than on data alone. From the point of view of AI, the focus on GENEOs corresponds to the rising interest in the so called “explainable deep learning” (Rudin, 2019; Carrieri et al., 2021; Hicks et al., 2021), which looks for methods and techniques that can be understood by humans.

GENEOs transform data according to two properties. First of all, they are equivariant with respect to the action of a given transformation group, i.e., they commute with such a group. Secondly, they do not increase the distance between data. This kind of regularity is frequently found in applications, since in several cases the operators we use are required to simplify the metric structure of data. We can obviously imagine particular applications where this condition is locally violated, but the usual long-term goal is to produce representations that are much simpler and more meaningful than the original data, thereby leading us to assume that the considered (compositions of any sufficiently long chain of) operators are non-expansive. This assumption is not only of use to simplify the information we have to manage, but it is also fundamental in the proof that the space of group equivariant non-expansive operators is compact (and hence finitely approximable), provided that the space of data is compact with respect to a suitable topology (Bergomi et al., 2019). This statement becomes false if we renounce non-expansivity.

The use of GENEOs is not limited to machine learning. Another important reason for the study of these operators follows from the relationship between GENEOs and TDA. We indeed know that TDA and Persistent Homology allow for a qualitative and efficient geometric study of the data space, but suffer from some important limitations, since Persistent Homology alone is not able to distinguish between some functions. Fortunately, the joint use of TDA and GENEOs overcomes this difficulty in the discrimination of data (Frosini, 2016; Frosini and Jabłoński, 2016; Bergomi et al., 2019). In other words, GENEOs are able to preserve information on the data that would have been lost through TDA alone.

Another interesting aspect of GENEOs is that we can also look at them as operators that change the pseudo-metrics we use in data comparison. If the real-valued functions φ_1_, φ_2_ represent the data we have to compare and a GENEO *F* is given, we can replace the max-norm distance ||φ_1_ − φ_2_||_∞_ with the new pseudo-metric $d^{\prime }\left(\varphi_{1},\varphi_{2}\right):=||F\left(\varphi_{1}\right)-F\left(\varphi_{2}\right)|{|}_{\infty }$. In this approach, *F* is not seen as a map that transforms the data we are considering, but as a new way of comparing data. We will see in section 6 that the availability of non-linear GENEOs can indeed produce more flexible pseudo-metrics.

Last but not least, a theory of GENEOs could be a relevant tool in the investigation of the role of internal conflicts in AI. We know that the availability of procedures that emulate intelligence opens the way to the appearance of contradictions, conflicts and unexpected behaviors (Frosini, 2009). This phenomenon cannot be ignored in the mathematical study of AI. The use of a precise geometric formalization of components in machine learning could be of great help in facing and analyzing this emerging problem.

However, the main reason for the research about GENEOs follows from a shift of interest from the spaces of data to the topological and geometric analysis of the spaces of observers of the data. This fact naturally leads us to the problem of the efficient approximation of observers. Such an approximation requires to make available large and dense sets of GENEOs, each one representing a possible data-observer interaction. Therefore, since non-linear interactions between observers and data are of great importance in applications, new techniques to build non-linear GENEOs are needed. The main contribution of this paper consists in introducing a new method to produce non-linear GENEOs through the concepts of symmetric function and permutant, thereby extending the procedure illustrated in Botteghi et al. (2020) for the building of linear GENEOs. In this way, we strictly expand the set of operators we can use in applications.

The concept of permutant comes into play when a set Φ of functions from a space *X* to ℝ and a group *G* of permutations of *X* are given. The set Φ represents the space of signals we are interested in, and is assumed to be preserved by right composition with elements of *G*. If two signals φ_1_, φ_2_ ∈ Φ are obtained from each other by right composition with an element *g* ∈ *G*, we say that they are equivalent with respect to *G*, just as happens when two images are considered equivalent if there exists an isometry changing one into the other. In this setting, a *permutant* is defined as a finite set *H* of Φ-preserving permutations of *X* that is stable under the conjugation action *h* ↦ *g* ◦ *h* ◦ *g*^−1^ of any element of *G* on *H* (Camporesi et al., 2018).

This paper shows that when a symmetric function and a permutant for the equivariance group *G* are available, we can easily build a (non-linear) GENEO with respect to *G* (section 3). This fact justifies the theoretical and practical importance of permutants. Our long-term purpose is the one of developing an effective theory for the approximation of observers and agents *via* GENEOs in a topological-geometrical setting, so extending the use of these operators in deep learning. While this goal is challenging, we think that our approach could lead to think of GENEOs as elementary components in the building of a new kind of neural networks. This idea is justified by at least two reasons. First of all, deep learning could benefit from using components that are guaranteed to be equivariant with respect to given groups of transformations and are grounded in a well founded topological theory, thereby allowing neural nets to save time in the learning process and to take advantage of techniques developed in TDA. Secondly, an engineering based on GENEOs would be much more transparent, because of the intrinsic interpretability of its components.

The reader could wonder why building GENEOs *via* permutants should be better than building them by other methods (for example by integrating on the equivariance group *G*). The key point is that in many applications some permutants exist, whose size is much smaller than the size of the equivariance group. In these cases, the approaches based on permutants can be much simpler than the ones based on *G*. We observe that permutants encode part of the information represented by the data equivalence expressed by *G*. Of course, by deciding to build GENEOs *via* permutants we implicitly accept to lose some information about such a data equivalence, and make a compromise between the computational complexity and the analytical power of the operators we are interested in. The reader can understand this tradeoff by thinking about the limit case given by a permutant containing just the identical permutation id of *X*. While the singleton {id} is indeed a (trivial) permutant, it does not give any information about the equivariance group *G* we are considering, since {id} is a permutant for *any* group of Φ-preserving permutations of *X*. However, if we consider a larger and larger set *H* of Φ-preserving permutations of *X*, the set of groups admitting *H* as a permutant becomes smaller and smaller. In other words, larger permutants make easier the identification of *G*.

This article is part of an extensive research on permutants. In Botteghi et al. (2020) it has been proved that each *linear* *G*-equivariant non-expansive operator can be produced by a weighted summation associated with a suitable weighted permutant, provided that the group *G* transitively acts on a finite signal domain. This paper opens the way to the research about the natural conjecture that each *non-linear* *G*-equivariant non-expansive operator can be produced (or at least well approximated) by applying our new technique to suitable symmetric functions and permutants, provided that the group *G* transitively acts on a finite signal domain. This probably non-trivial problem will be attacked in following papers, grounding on the results obtained in this article.

The outline of the paper is as follows. In section 2, we recall the main definitions in our mathematical setting. In Section 3, we show how to associate a group equivariant operator (GEO) with a symmetric function. Section 4 is devoted to the approximation of a generic continuous symmetric function by a polynomial in the elementary symmetric functions, and in section 5, we finally show how to associate a GENEO with such a polynomial. Section 6 highlights the benefits of our approach.

For more details and proofs about the results and concepts illustrated in section 2 we refer the interested reader to the papers (Frosini, 2016; Frosini and Jabłoński, 2016; Frosini and Quercioli, 2017; Camporesi et al., 2018; Bergomi et al., 2019). The other sections present our new results about the construction of non-linear GENEOs *via* symmetric functions and permutants.

## 2. Mathematical Setting

Let *X* be a non-empty set and consider a non-empty, compact subspace Φ of the normed vector space $\left({\mathbb{R}}_{b}^{X},||\cdot |{|}_{\infty }\right)$, where ${\mathbb{R}}_{b}^{X}$ is the set of all bounded real-valued functions with domain *X*, and $\varphi_{\infty }:=\sup_{x\in X}|\varphi \left(x\right)|$. We can think of the functions in Φ as the data, i.e., the measurements provided by our measuring instruments (or by any operator), and of *X* as the space where the measurements are made. Sometimes the functions in Φ are also referred as **admissible filtering functions** or **admissible signals**. We now recall the usual setting for the introduction of group equivariant non-expansive operators. We endow Φ with the topology induced by the uniform convergence distance


$$D_{\Phi }\left(\varphi_{1},\varphi_{2}\right)\;:={\left\|\varphi_{1}-\varphi_{2}\right\|}_{\infty }.$$

At this stage, *X* is only a set. We endow *X* with the topology induced by the pseudo-metric


$$D_{X}\left(x_{1},x_{2}\right)\;:=\underset{\varphi \in \Phi }{\sup}|\varphi \left(x_{1}\right)-\varphi \left(x_{2}\right)|.$$

The idea behind this definition is that two points *x*_1_, *x*_2_ ∈ *X* are considered different only if they are taken to different values by at least one admissible filtering function.

We recall that a pseudo-metric space is a generalization of a metric space in which the distance between two distinct points can be zero. Moreover, a function *f* from a pseudo-metric space (*P*_1_, *d*_1_) to a pseudo-metric space (*P*_2_, *d*_2_) is called **non-expansive** if


$$d_{2}\left(f\left(x\right),f\left(y\right)\right)\leq d_{1}\left(x,y\right)$$

for every *x, y* ∈ *P*_1_.

**Remark 2.1**. *Every function φ* ∈ Φ *is non-expansive with respect to the pseudo-metric *D*_*X*_ on *X* and the Euclidean metric on* ℝ*. Therefore, each function φ* ∈ Φ *is continuous with respect to these topologies*.

Since Φ is compact, the topology induced by the pseudo-metric *D*_*X*_ coincides with the initial topology τ_*in*_ on *X* with respect to Φ (see Theorem 2.1 in Bergomi et al., 2019, Supplementary Methods). We recall that the initial topology is the coarsest topology on *X* which makes each function in Φ continuous. Moreover, the compactness of Φ implies that if *X* is complete then it is also compact (see Theorem 2.2 in Bergomi et al., 2019, Supplementary Methods). In this work, we assume that *X* is complete, and therefore compact with respect to the topology induced by *D*_*X*_. The image of *X* through the filtering functions is denoted by Im(Φ) and is defined as


Im(Φ)={φ(x) s.t. φ∈Φ,x∈X}.

The following result will be of use in section 5.

**Proposition 2.2**. *If *X* and* Φ *are compact,* Im(Φ) *is compact with respect to the Euclidean topology*.

*Proof*. Let us consider the function γ : Φ × *X* → ℝ such that γ(φ, *x*) : = φ(*x*). The space Φ × *X* is compact with respect to the product topology, which we recall is induced by the sum pseudo-distance. Since the continuous image of a compact is compact, γ(Φ × *X*) = Im(Φ), and every non-expansive function is continuous, it is sufficient to prove that γ is non-expansive. Given φ_1_, φ_2_ ∈ Φ and *x*_1_, *x*_2_ ∈ *X*, we have that


$$\begin{array}{l} \left|\gamma \left(\varphi_{1},x_{1}\right)-\gamma \left(\varphi_{2},x_{2}\right)|=|\varphi_{1}\left(x_{1}\right)-\varphi_{2}\left(x_{2}\right)\right|\\ \;=|\varphi_{1}\left(x_{1}\right)-\varphi_{1}\left(x_{2}\right)+\varphi_{1}\left(x_{2}\right)-\varphi_{2}\left(x_{2}\right)|\\ \;\leq \underset{\varphi \in \Phi }{\sup}|\varphi \left(x_{1}\right)-\varphi \left(x_{2}\right)|\\ \;+\underset{x\in X}{\sup}|\varphi_{1}\left(x\right)-\varphi_{2}\left(x\right)|\\ \;=D_{X}\left(x_{1},x_{2}\right)+D_{\Phi }\left(\varphi_{1},\varphi_{2}\right). \end{array}$$

We have proved that γ is non-expansive, and therefore Im(Φ) is compact.

**Definition 2.3**. Chachólski et al. (2020) *A* **Φ**-***operation** is a function *g* : *X* → *X* such that, for every φ* ∈ Φ, *the composition φ* ◦ *g also belongs to* Φ.

**Definition 2.4**. *A* Φ-operation *g is **invertible** if there is a* Φ-*operation *h* such that *g* ◦ *h* = *h* ◦ *g* = id_*X*_*.

We denote the collection of all invertible Φ-operations by Aut_Φ_(*X*). In other words,


$$\begin{array}{l} {\text{Aut}}_{\Phi }\left(X\right)=\{g\;:\;X\to X|g\text{ is a bijection, and }\varphi ◦g,\\ \;\varphi ◦g^{-1}\in \Phi \text{for every }\varphi \in \Phi \}. \end{array}$$

We note that Aut_Φ_(*X*) is a group with respect to the usual composition operation.

**Definition 2.5**. *A **perception pair** is an ordered pair* (Φ, *G*) *where* $\Phi \subseteq {\mathbb{R}}_{b}^{X}$ *and G is a subgroup of* Aut_Φ_(*X*).

As an example, (Φ, Aut_Φ_(*X*)) is always a perception pair.

**Remark 2.6**. *When a perception pair* (Φ, *G*) *is given, each element g* ∈ *G acts on the set* Φ *by right composition, taking each function φ* ∈ Φ *to the function* φ ◦ *g*.

### 2.1. Group Equivariant Non-Expansive Operators

**Definition 2.7**. *Let us consider two perception pairs* (Φ, *G*), (Ψ, *H*) *and a homomorphism T* : *G* → *H*. *Each map F* : Φ → Ψ *such that F is T-equivariant (i.e., F(φ* ◦ *g*) = *F*(φ) ◦ *T*(*g*) *for every* φ ∈ Φ, *g* ∈ *G*) *is called a **Group Equivariant Operator (GEO)** with respect to T*.

**Definition 2.8**. *Let us consider two perception pairs* (Φ, *G*), (Ψ, *H*) *and a homomorphism T* : *G* → *H*. *Each map F* : Φ → Ψ *such that F is T-equivariant (i.e.*, *F*(φ ◦ *g*) = *F*(φ) ◦ *T*(*g*) *for every* φ ∈ Φ, *g* ∈ *G*) *and non-expansive (i.e., ||F*(φ) − *F*(ψ)||_∞_ ≤ φ − ψ_∞_ *for every* φ, ψ ∈ Φ) *is called a **Group Equivariant Non-Expansive Operator (GENEO)** with respect to T*.

After fixing two perception pairs (Φ, *G*), (Ψ, *H*) and a homomorphism *T* : *G* → *H*, we will use the symbol $F_{T}^{\text{all}}$ to denote the collection of all GENEOs with respect to *T* between such perception pairs. We endow $F_{T}^{\text{all}}$ with the topology induced by the metric $D_{\text{GENEO}}\left(F_{1},F_{2}\right):=\sup_{\varphi \in \Phi }D_{\Phi }\left(F_{1}\left(\varphi \right),F_{2}\left(\varphi \right)\right)$. For a more in-depth study of the GENEO topology, we refer the reader to Bergomi et al. (2019). We stress that the non-expansivity of the operators is pivotal for two reasons. The first reason is that we want our operators to simplify the data metric, i.e., not to introduce complexity into the data. The second reason is that non-expansivity allows us to prove the compactness of the space $F_{T}^{\text{all}}$, provided that Φ and Ψ are compact with respect to the distances *D*_Φ_, *D*_Ψ_ (see Theorem 7 in Bergomi et al., 2019). If we remove the assumption that our operators are non-expansive, this property of compactness does not hold anymore. As an example, let Φ = Ψ be equal to the set of all constant functions from ℝ to [0, 1], and *G* = *H* be the trivial group containing just the identity permutation of ℝ. We observe that Φ, *X* = ℝ and *G* are compact with respect to the topologies we have defined on them. Let us now consider the sequence (*F*_*n*_) of GEOs from Φ to Φ with respect to the identity homomorphism id_*G*_ : *G* → *G*, defined by setting $F_{n}\left(\varphi \right):=\varphi^{n}$ for every function φ ∈ Φ and every positive integer *n*. It is easy to check that $\underset{n\to \infty }{\lim}D_{\text{GENEO}}\left(F_{m},F_{n}\right)=1$ for every positive integer *m*, and hence the sequence (*F*_*n*_) does not admit any converging subsequence. This implies that the space of all GEOs from Φ to Φ with respect to id_*G*_ is not compact. The compactness of $F_{T}^{\text{all}}$ is a key property in applications, since it guarantees that such a space can be approximated by a finite set.

If *G* = *H* and *T* = id_*G*_, we can say that $F:\Phi \to {\mathbb{R}}_{b}^{X}$ is a *G*-equivariant map. From now on, we will make these assumptions, and use the terms GEO and GENEO with reference to this setting.

### 2.2. Permutants

**Definition 2.9**. *Let *S*_*X*_ be the set of permutations of *X*. For each *g* ∈ *G*, the map *c*_*g*_ : *S*_*X*_ → *S*_*X*_ taking each *s* ∈ *S*_*X*_ to *g* ◦ *s* ◦ *g**^−1^ *is called the **conjugation action** of *g* ∈ *G* on *S*_*X*_. For every subset *H* of *S*_*X*_, we denote the set *c*_*g*_(*H*) by the symbol *gHg**^−1^.

**Definition 2.10**. Camporesi et al. (2018) *A finite set H* ⊆ Aut_Φ_(*X*) *is called a **permutant** for *G* if either *H* = ∅ or *gHg**^−1^ = *H for every g ∈ G*.

**Remark 2.11**. *In general, a permutant is not a normal subgroup of *G*. Indeed we require neither that *H* is a group nor that *H* is a subset of *G*. We observe that the sets* ∅ *and* {id_*X*_} *are trivial permutants for any subgroup *G* of* Aut_Φ_(*X*). *Both *G* and* Aut_Φ_(*X*) *are also permutants for *G*, provided that they are finite groups*.

**Example 2.12**. *Let* Φ *be the set of all functions* φ : *X* = *S*^1^ = {(*x, y*) ∈ ℝ^2^|*x*^2^ + *y*^2^ = 1} → [0, 1] *that are non-expansive with respect to the Euclidean distances on* *S*^1^ *and* [0, 1]. *Let us consider the group *G* of all isometries of* ℝ. *If *h* is the clockwise rotation of ℓ radians for a fixed ℓ* ∈ ℝ, *then the set *H** = {*h, h*^−1^} *is a permutant for *G**.

**Example 2.13**. *Let* Φ *be the set of all functions* φ : *X* = *S*^1^ = {(*x, y*) ∈ ℝ^2^|*x*^2^ + *y*^2^ = 1} → [0, 1] *that are non-expansive with respect to the Euclidean distances on S*^1^ *and* [0, 1]. *Let *G* be the group generated by the reflection with respect to the axis x* = 0. *If ρ is the clockwise rotation of π/2 around the origin* (0, 0), *then the set* $H=\left\{{\text{id}}_{S^{1}},\rho ,\rho^{2},\rho^{3}\right\}$ *is a permutant for G*.

**Example 2.14**. *Let us consider the set *X* of the vertices of a cube in* ℝ^3^, *and assume that* Φ *is the set of all functions from X to* [0, 1]. *Let *G* be the group of the orientation-preserving isometries of* ℝ^3^ *that take X to *X*. Let π_1_, π_2_, π_3_ be the three planes that contain the center of mass of *X* and are parallel to a face of the cube. Let h*_*i*_ : *X* → *X be the orthogonal symmetry with respect to π_*i*_, for i* ∈ {1, 2, 3}. *We have that the set H* = {*h*_1_, *h*_2_, *h*_3_} *is an orbit under the conjugation action of *G* on* Aut_Φ_(*X*), *and therefore a permutant for *G**.

**Remark 2.15**. *If the group *G* is Abelian, every finite subset of *G* is a permutant for *G*, since the conjugation action of *G* on* Aut_Φ_(*X*) *is just the identity*.

**Remark 2.16**. *In this section, the symbol* || · ||_∞_ *has been used to denote the max-norm of functions. With a slight abuse of notation, in the rest of the paper such a symbol will be also used to denote the max-norm of points of* ℝ^*m*^, *i.e.,* $\left||\left(\alpha_{1},\ldots ,\alpha_{m}\right)|{|}_{\infty }:=\underset{1\leq i\leq m}{\max}|\alpha_{i}\right|$.

## 3. Building GEOs From Symmetric Functions

**Definition 3.1**. *Let *C* be a symmetric subset of* ℝ^*n*^, *i.e., a subset *C* such that π(*C*) = *C* for every permutation π of the coordinates. A function f* : *C* → ℝ *is said to be **symmetric on** **C** if its value is the same no matter the order of its arguments. That is*,


$$f\left(a_{1},\ldots ,a_{n}\right)=f\left(a_{\pi \left(1\right)},\ldots ,a_{\pi \left(n\right)}\right)$$

*for every* (*a*_1_, …, *a*_*n*_) ∈ *C and every permutation π of the set* {1, …, *n*}.

**Proposition 3.2**. *Let *f* be a continuous real-valued symmetric function defined on a compact symmetric subset *K* of* ℝ^*n*^. *Then *f* is the restriction of a continuous real-valued symmetric function* $\overset{-}{f}$ *defined on* ℝ^*n*^.

*Proof*. The Tietze Extension Theorem (Dugundji, 1966) implies that *f* can be extended to a continuous function $\hat{f}:{\mathbb{R}}^{n}\to \mathbb{R}$. If *S*_*n*_ is the symmetric group over the set {1, …, *n*}, we can easily check that the function $\overset{-}{f}\left(a_{1},\ldots ,a_{n}\right):=\frac{1}{n!}\underset{\pi \in S_{n}}{\sum }\hat{f}\left(a_{\pi \left(1\right)},\ldots ,a_{\pi \left(n\right)}\right)$ has the wanted property.

Proposition 3.2 guarantees that the concept of continuous real-valued symmetric function defined on a compact symmetric subset *K* of ℝ^*n*^ coincides with the concept of restriction to *K* of a continuous real-valued symmetric function defined on ℝ^*n*^.

Let $S:{\mathbb{R}}^{n}\to \mathbb{R}$ be a symmetric function. If $H={\left\{h_{i}\right\}}_{i=1}^{n}$ is a non-empty permutant for *G* ⊆ Aut_Φ_(*X*), then we can define an operator $S_{H}:\Phi \to {\mathbb{R}}_{b}^{X}$ by setting, for any φ ∈ Φ,


$$S_{H}\left(\varphi \right)\;:=S\left(\varphi ◦h_{1},\ldots ,\varphi ◦h_{n}\right),$$

where $S\left(\varphi ∙h_{1},\ldots ,\varphi ∙h_{n}\right)\left(x\right):=S\left(\left(\varphi ∙h_{1}\right)\left(x\right),\ldots ,\left(\varphi ∙h_{n}\right)\left(x\right)\right)$ for every *x* ∈ *X*.

**Proposition 3.3**. *If* $S:{\mathbb{R}}^{n}\to \mathbb{R}$ *is a symmetric function and G* ⊆ Aut_Φ_(*X*), *then* $S_{H}$ *is a GEO from* Φ *to* ${\mathbb{R}}_{b}^{X}$ *with respect to the identity homomorphism* id_*G*_ : *G* → *G*.

*Proof*. For every *g* ∈ *G*, it holds that


$$\begin{array}{l} S_{H}\left(\varphi ◦g\right)=S\left(\varphi ◦g◦h_{1},\ldots ,\varphi ◦g◦h_{n}\right)\\ \;=S\left(\left(\varphi ◦h_{\pi_{g}\left(1\right)}\right)◦g,\ldots ,\left(\varphi ◦h_{\pi_{g}\left(n\right)}\right)◦g\right)\\ \;=S\left(\left(\varphi ◦h_{1}\right)◦g,\ldots ,\left(\varphi ◦h_{n}\right)◦g\right)\\ \;=S\left(\varphi ◦h_{1},\ldots ,\varphi ◦h_{n}\right)◦g\\ \;=S_{H}\left(\varphi \right)◦g, \end{array}$$

where π_*g*_ is the permutation such that $g∙h_{i}∙g^{-1}=h_{\pi_{g}\left(i\right)}$, that is *g* ◦ *h*_*i*_ = *h*_π_*g*_(*i*)_ ◦ *g*. Therefore, $S_{H}\left(\varphi ∙g\right)=S_{H}\left(\varphi \right)∙g$, and hence $S_{H}$ is a GEO.

**Corollary 3.4**. *If* $S:{\mathbb{R}}^{n}\to \mathbb{R}$ *is a symmetric function and its restriction to* Im(Φ)^*n*^ *is non-expansive, then* $S_{H}$ *is a GENEO from* Φ *to* ${\mathbb{R}}_{b}^{X}$ *with respect to* id_*G*_.

*Proof*. It is sufficient to prove the non-expansivity of $S_{H}$ with respect to the max-norms on Φ and ${\mathbb{R}}_{b}^{X}$, since the group equivariance is already granted by Proposition 3.3. If φ, ψ ∈ Φ, then for every *x* ∈ *X*


$$\begin{array}{l} \left|\left(S_{H}\left(\varphi \right)\right)\left(x\right)-\left(S_{H}\left(\psi \right)\right)\left(x\right)\right|\\ =|S\left(\varphi ◦h_{1},\ldots ,\varphi ◦h_{n}\right)\left(x\right)-S\left(\psi ◦h_{1},\ldots ,\psi ◦h_{n}\right)\left(x\right)|\\ \leq {\left\|\left(\left(\varphi ◦h_{1}\right)\left(x\right)-\left(\psi ◦h_{1}\right)\left(x\right),\ldots ,\left(\varphi ◦h_{n}\right)\left(x\right)-\left(\psi ◦h_{n}\right)\left(x\right)\right)\right\|}_{\infty }\\ =\underset{1\leq i\leq n}{\max}|\left(\varphi ◦h_{i}\right)\left(x\right)-\left(\psi ◦h_{i}\right)\left(x\right)|\\ \leq \underset{1\leq i\leq n}{\max}{\left\|\varphi ◦h_{i}-\psi ◦h_{i}\right\|}_{\infty }\\ ={\left\|\varphi -\psi \right\|}_{\infty }. \end{array}$$

In conclusion, ${{||S}_{H}\left(\varphi \right)-S_{H}\left(\psi \right)||}_{\infty }\leq {||\varphi -\psi ||}_{\infty }$ and $S_{H}$ is a GENEO.

So far we have shown how to construct GEOs associated with a symmetric function. These operators are actually GENEOs if the function they are associated with is non-expansive. In the next sections, we will show how to adapt this concept to build GENEOs even in the presence of symmetric functions that are not non-expansive.

We stress that our approach requires no integration over the (possibly infinite and large) group *G*, but just the availability of a permutant and the computation of a symmetric function. This approach generalizes the method introduced in Camporesi et al. (2018), concerning the symmetric function $S\left(a_{1},\ldots ,a_{n}\right)=\frac{1}{n}\overset{n}{\underset{i=1}{\sum }}a_{i}$.

## 4. Approximating Symmetric Functions With Symmetric Polynomials

Let us now explore the concept of approximation of symmetric functions by symmetric polynomials. For more details, we refer the reader to Davidson and Donsig (2009), Blum-Smith and Coskey (2017). In the sequel, we will denote the symmetric group over the set {1, …, *n*} as *S*_*n*_. Let *K* be a compact metric space, and C(K) be the vector space of continuous real-valued functions on *K*. With a slight abuse of notation, in the following we will confuse each polynomial with the function it represents, restricted to the domain we are considering. Furthermore, if *I* is a finite subset of ℕ^*n*^, we will say that a polynomial $\underset{\left(k_{1},\ldots ,k_{n}\right)\in I}{\sum }c_{k_{1},\ldots ,k_{n}}y_{1}^{k_{1}}\cdot \ldots \cdot y_{n}^{k_{n}}$ is **symmetric** if π(*I*) = *I*, and *c*_*k*_1_, …, *k*_*n*__ = *c*_π(*k*_1_), …, π(*k*_*n*_)_ for every multi-index (*k*_1_, …, *k*_*n*_) ∈ *I* and every permutation π ∈ *S*_*n*_.

**Definition 4.1**. Davidson and Donsig (2009) *A subset *A* of C(K) is an **algebra** if it is a vector subspace of C(K) that is closed under multiplication (i.e., if *f, g* ∈ *A* then *f* · *g* ∈ *A*). A set *S* of functions on *K* **separates points** if for each pair of points *s, t* ∈ *K* there is a function *f* ∈ *S* such that f*(*s*) ≠ *f*(*t*). *A set *S* of functions on *K* **vanishes** at *s* ∈ *K* if f*(*s*) = 0 *for all *f* ∈ S*.

**Theorem 4.2 (Stone - Weierstrass Theorem)**. Davidson and Donsig (2009) *An algebra *A* of continuous real-valued functions on a compact metric space *K* that separates points and does not vanish at any point is dense in* C(K) *with respect to the max-norm referred to the domain *K**.

**Corollary 4.3**. Davidson and Donsig (2009) *Let *K* be a compact subset of* ℝ^*n*^. *The algebra of all polynomials p*(*y*_1_, …, *y*_*n*_) *in *n* variables is dense in* C(K) *with respect to the max-norm referred to the domain K*.

This theorem allows us to approximate a continuous symmetric function S:K→ℝ by a polynomial with arbitrary accuracy, provided that *K* is a compact subset of ℝ^*n*^. However, this is not exactly what we need, as we would like such a polynomial to be symmetric. This can be obtained by a symmetrization of the previously found polynomial, as shown by the next proposition, which proves that any symmetric continuous function on a compact and symmetric domain can be approximated with arbitrary precision by a symmetric polynomial.

**Proposition 4.4**. *Let *K* be a compact subset of* ℝ^*n*^, *verifying the property π(*K*) = *K* for every π ∈ *S*_*n*_. If* $S_{|K}:K\to \mathbb{R}$ *is the restriction to *K* of a continuous symmetric function* $S:{\mathbb{R}}^{n}\to \mathbb{R}$ *and* || · ||_∞_ *is the max-norm referred to the domain *K*, then for every ε* > 0 *there exists a symmetric polynomial *q* in *n* variables such that* ${||S_{|K}-q_{|K}||}_{\infty }\leq \varepsilon$.

*Proof*. From Corollary 4.3 it follows that there exists a polynomial *p* : ℝ^*n*^ → ℝ such that ${{||S}_{|K}-p_{|K}||}_{\infty }\leq \varepsilon$. Let us now define the symmetric polynomial $q\left(a_{1},\ldots ,a_{n}\right):=\frac{1}{n!}\underset{\pi \in S_{n}}{\sum }p\left(a_{\pi \left(1\right)},\ldots ,a_{\pi \left(n\right)}\right)$. If *a* = (*a*_1_, …, *a*_*n*_) ∈ *K*, we define *a*_π_ = (*a*_π(1)_, …, *a*_π(*n*)_) for every permutation π ∈ *S*_*n*_. Then


$$\begin{array}{l} {\left\|S_{|K}-q_{|K}\right\|}_{\infty }=\underset{a\in K}{\max}|S\left(a\right)-q\left(a\right)|\\ \;=\underset{a\in K}{\max}\left|S\left(a\right)-\frac{1}{n!}\underset{\pi \in S_{n}}{\sum }p\left(a_{\pi }\right)\right|\\ \;=\underset{a\in K}{\max}\left|\frac{1}{n!}\underset{\pi \in S_{n}}{\sum }S\left(a\right)-\frac{1}{n!}\underset{\pi \in S_{n}}{\sum }p\left(a_{\pi }\right)\right|\\ \;\leq \frac{1}{n!}\underset{a\in K}{\max}\underset{\pi \in S_{n}}{\sum }|S\left(a\right)-p\left(a_{\pi }\right)|\\ \;=\frac{1}{n!}\underset{a\in K}{\max}\underset{\pi \in S_{n}}{\sum }|S\left(a_{\pi }\right)-p\left(a_{\pi }\right)|\\ \;=\frac{1}{n!}\underset{\pi \in S_{n}}{\sum }\underset{a\in K}{\max}|S\left(a_{\pi }\right)-p\left(a_{\pi }\right)|\\ \;=\frac{1}{n!}\underset{\pi \in S_{n}}{\sum }\underset{a\in K}{\max}|S\left(a\right)-p\left(a\right)|\\ \;=\frac{1}{n!}\underset{\pi \in S_{n}}{\sum }{\left\|S_{|K}-p_{|K}\right\|}_{\infty }\\ \;\leq \frac{1}{n!}\underset{\pi \in S_{n}}{\sum }\varepsilon =\varepsilon . \end{array}$$

**Definition 4.5**. *The **elementary symmetric polynomials** in the *n* variables a*_1_, …, *a*_*n*_, *also called **elementary symmetric functions**, are defined as*:


$$\begin{array}{l} \sigma_{1}\;:=a_{1}+\ldots +a_{n}\\ \sigma_{2}\;:=a_{1}\cdot a_{2}+a_{1}\cdot a_{3}+\ldots +a_{n-1}\cdot a_{n}=\underset{1\leq i<j\leq n}{\sum }a_{i}\cdot a_{j}\\ \vdots \\ \sigma_{r}\;:=\underset{1\leq i_{1}<i_{2}<\cdots <i_{r}\leq n}{\sum }a_{i_{1}}\cdot a_{i_{2}}\cdot \ldots \cdot a_{i_{r}}=\underset{1\leq i_{1}<i_{2}<\cdots <i_{r}\leq n}{\sum }\overset{i_{r}}{\underset{j=i_{1}}{\prod }}a_{j}\\ \vdots \\ \sigma_{n}\;:=a_{1}\cdot a_{2}\cdot \ldots \cdot a_{n}. \end{array}$$

We now recall an important result in the theory of symmetric polynomials:

**Theorem 4.6. (Fundamental Theorem on Symmetric Polynomials)**. Blum-Smith and Coskey (2017) Any symmetric polynomial in *n* variables *a*_1_, …, *a*_*n*_ is representable in a unique way as a polynomial in the elementary symmetric polynomials σ_1_, …, σ_*n*_.

**Remark 4.7**. *It is a well know fact (see Rao, 2005; Davidson and Donsig, 2009; Blum-Smith and Coskey, 2017) that the proofs of Theorems 4.2 and 4.6 are constructive. This means that, if *K* is a compact symmetric subset of* ℝ^*n*^, *and the restriction* $S_{|K}$ *of a continuous symmetric function* $S:{\mathbb{R}}^{n}\to \mathbb{R}$ *is given, we are able to effectively approximate* $S_{|K}$ *with an error less than ε by the restriction to *K* of an explicitly defined polynomial in the elementary symmetric functions*.

In conclusion, if an equivariance group *G* is chosen and a GEO *F* is built by applying Proposition 3.3 to the continuous symmetric function $S:{\mathbb{R}}^{n}\to \mathbb{R}$, we can approximate *F* in the following way, provided that *X* and Φ are compact. First of all, we can approximate the continuous function S by a polynomial *p* : ℝ^*n*^ → ℝ, with an arbitrarily small error ε on the symmetric set Im(Φ)^*n*^, which is guaranteed to be compact by Proposition 2.2. Then, we can consider the symmetric polynomial $q\left(a_{1},\ldots ,a_{n}\right):=\frac{1}{n!}\underset{\pi \in S_{n}}{\sum }p\left(a_{\pi \left(1\right)},\ldots ,a_{\pi \left(n\right)}\right)$. Finally, we can consider the GEO *F*′ defined by setting $F^{\prime }\left(\varphi \right):=q\left(\varphi ∙h_{1},\ldots ,\varphi ∙h_{n}\right)$ for every φ ∈ Φ. Since *H* ⊆ Aut_Φ_(*X*), $||F\left(\varphi \right)-F^{\prime }\left(\varphi \right)|{|}_{\infty }=\underset{x\in X}{\max}|S\left(\varphi \left(h_{1}\left(x\right)\right),\ldots ,\varphi \left(h_{n}\left(x\right)\right)\right)-q\left(\varphi \left(h_{1}\left(x\right)\right),\ldots ,\varphi \left(h_{n}\left(x\right)\right)\right)|\leq ||S_{|{\text{Im}\left(\Phi \right)}^{n}}-q_{|{\text{Im}\left(\Phi \right)}^{n}}|{|}_{\infty }\leq \varepsilon$for any φ ∈ Φ, and hence the operator *F*′ can be chosen arbitrarily close to *F*.

## 5. Building GENEOs From Polynomials in the Elementary Symmetric Functions

Proposition 2.2 shows that Im(Φ) is compact. Moreover, the equality π (Im(Φ)^*n*^) = Im(Φ)^*n*^ trivially holds for every π ∈ *S*_*n*_. Therefore, Proposition 4.4 and the Fundamental Theorem on Symmetric Polynomials guarantee that the restriction to Im(Φ)^*n*^ of any continuous symmetric functions can be approximated arbitrarily well by the restriction to Im(Φ)^*n*^ of a polynomial in the elementary symmetric functions, defined as


$$\tilde{S}\left(a_{1},\ldots ,a_{n}\right)=\overset{m_{1}}{\underset{k_{1}=0}{\sum }}\ldots \overset{m_{n}}{\underset{k_{n}=0}{\sum }}c_{k_{1},\ldots ,k_{n}}\overset{n}{\underset{i=1}{\prod }}\sigma_{i}^{k_{i}}\left(a_{1},\ldots ,a_{n}\right),$$

where *m*_*i*_ ∈ ℕ for every *i* ∈ {1, …, *n*}, *c*_*k*_1_, …, *k*_*n*__ ∈ ℝ for every *k*_1_ ∈ {0, …, *m*_1_}, …, *k*_*n*_ ∈ {0, …, *m*_*n*_} and σ_*i*_ is the *i*-th elementary symmetric polynomial for every *i* ∈ {1, …, *n*}. From Proposition 3.3, we already know that the associated operator is a GEO. We can indeed obtain a GENEO by applying Corollary 3.4 to a suitable multiple of $\tilde{S}$. In the sequel, we will need the following constants:


(5.1) $$\begin{array}{r} M_{{\text{Im}\left(\Phi \right)}^{n}}\;:=\underset{\alpha \in {\text{Im}\left(\Phi \right)}^{n}}{\max}{\left\|\alpha \right\|}_{\infty }=\underset{\varphi \in \Phi }{\max}{\left\|\varphi \right\|}_{\infty } \end{array}$$

(5.2) M1 :=max1≤i≤n{ki(ni)kiiMIm(Φ)niki-1}

(5.3) M2 :=max1≤i≤n{(ni)kiMIm(Φ)niki}n-1

(5.4) $$\begin{array}{r} C=n\overset{m_{1}}{\underset{k_{1}=0}{\sum }}\ldots \overset{m_{n}}{\underset{k_{n}=0}{\sum }}|c_{k_{1},\ldots ,k_{n}}|M_{1}M_{2}, \end{array}$$

Let us consider a non-empty permutant $H={\left\{h_{i}\right\}}_{i=1}^{n}$ for *G* ⊆ Aut_Φ_(*X*). We can define an operator ${\hat{S}}_{H}:\Phi \to {\mathbb{R}}_{b}^{X}$ by setting


$${\hat{S}}_{H}\left(\varphi \right)\;:=\frac{1}{C}\tilde{S}\left(\varphi ◦h_{1},\ldots ,\varphi ◦h_{n}\right)$$

for any φ ∈ Φ, where $\tilde{S}\left(\varphi ∙h_{1},\ldots ,\varphi ∙h_{n}\right)\left(x\right):=\tilde{S}\left(\left(\varphi ∙h_{1}\right)\left(x\right),\ldots ,\left(\varphi ∙h_{n}\right)\left(x\right)\right)$ for every *x* ∈ *X* and *C* is the constant defined in (5.4).

**Theorem 5.1**. *If* $\tilde{S}$ *is a polynomial in the *n* elementary symmetric functions, then* ${\hat{S}}_{H}$ *is a GENEO from* Φ *to* ${\mathbb{R}}_{b}^{X}$ *with respect to* id_*G*_.

*Proof*. The thesis immediately follows from Corollary 3.4, once it is proved that the restriction of $\frac{1}{C}\tilde{S}$ to Im(Φ)^*n*^ is non-expansive. For every $\alpha =\left(\alpha_{1},\ldots ,\alpha_{n}\right),\beta =\left(\beta_{1},\ldots ,\beta_{n}\right)\in {\text{Im}\left(\Phi \right)}^{n}$, by applying Lemma 1.5 in Appendix 1, we have that


$$\begin{array}{l} \left|\frac{1}{C}\tilde{S}\left(\alpha \right)-\frac{1}{C}\tilde{S}\left(\beta \right)\right|\\ =|\frac{1}{C}\overset{m_{1}}{\underset{k_{1}=0}{\sum }}\ldots \overset{m_{n}}{\underset{k_{n}=0}{\sum }}c_{k_{1},\ldots ,k_{n}}\overset{n}{\underset{i=1}{\prod }}\sigma_{i}^{k_{i}}\left(\alpha \right)\\ -\frac{1}{C}\overset{m_{1}}{\underset{k_{1}=0}{\sum }}\ldots \overset{m_{n}}{\underset{k_{n}=0}{\sum }}c_{k_{1},\ldots ,k_{n}}\overset{n}{\underset{i=1}{\prod }}\sigma_{i}^{k_{i}}\left(\beta \right)|\\ \leq \frac{1}{C}\overset{m_{1}}{\underset{k_{1}=0}{\sum }}\ldots \overset{m_{n}}{\underset{k_{n}=0}{\sum }}|c_{k_{1},\ldots ,k_{n}}|\left|\overset{n}{\underset{i=1}{\prod }}\sigma_{i}^{k_{i}}\left(\alpha \right)-\overset{n}{\underset{i=1}{\prod }}\sigma_{i}^{k_{i}}\left(\beta \right)\right|\\ \leq \frac{1}{C}\overset{m_{1}}{\underset{k_{1}=0}{\sum }}\ldots \overset{m_{n}}{\underset{k_{n}=0}{\sum }}|c_{k_{1},\ldots ,k_{n}}|n{\left\|\alpha -\beta \right\|}_{\infty }M_{1}M_{2}\\ =\left(\frac{1}{C}n\overset{m_{1}}{\underset{k_{1}=0}{\sum }}\ldots \overset{m_{n}}{\underset{k_{n}=0}{\sum }}|c_{k_{1},\ldots ,k_{n}}|M_{1}M_{2}\right){\left\|\alpha -\beta \right\|}_{\infty }\\ ={\left\|\alpha -\beta \right\|}_{\infty }. \end{array}$$

We have shown that $\frac{1}{C}\tilde{S}$ is non-expansive and therefore the associated operator ${\hat{S}}_{H}$ is a GENEO.     □

**Example 5.2**. *Let us consider the setting of Example 2.12 and the polynomial* $\tilde{S}\left(a_{1},a_{2}\right)=-\sigma_{1}\left(a_{1},a_{2}\right)+\sigma_{2}^{2}\left(a_{1},a_{2}\right)-3\sigma_{1}\left(a_{1},a_{2}\right)\sigma_{2}\left(a_{1},a_{2}\right)=-a_{1}-a_{2}+a_{1}^{2}a_{2}^{2}-3a_{1}^{2}a_{2}-3a_{1}a_{2}^{2}$. *Then the operator*


$$\begin{array}{l} {\hat{S}}_{H}\left(\varphi \right)=\frac{1}{40}(-\left(\varphi ◦h\right)-\left(\varphi ◦h^{-1}\right)+\left(\varphi^{2}◦h\right)\cdot \left(\varphi^{2}◦h^{-1}\right)\\ \;-3\left(\varphi^{2}◦h\right)\cdot \left(\varphi ◦h^{-1}\right)-3\left(\varphi ◦h\right)\cdot \left(\varphi^{2}◦h^{-1}\right)) \end{array}$$

*is a GENEO*.

**Example 5.3**. *Let us consider the setting of Example 2.13 and the polynomial* $\tilde{S}\left(a_{1},a_{2},a_{3},a_{4}\right)=\sigma_{1}\left(a_{1},a_{2},a_{3},a_{4}\right)+\sigma_{4}\left(a_{1},a_{2},a_{3},a_{4}\right)=a_{1}+a_{2}+a_{3}+a_{4}+a_{1}a_{2}a_{3}a_{4}$. *In this case, the operator*


$$\begin{array}{l} {\hat{S}}_{H}\left(\varphi \right)=\frac{1}{1040}(\varphi +\left(\varphi ◦\rho \right)+\left(\varphi ◦\rho^{2}\right)+\left(\varphi ◦\rho^{3}\right)\\ \;+\varphi \cdot \left(\varphi ◦\rho \right)\cdot \left(\varphi ◦\rho^{2}\right)\cdot \left(\varphi ◦\rho^{3}\right)) \end{array}$$

*is a GENEO*.

**Example 5.4**. *Let us consider the setting of Example 2.14 and the polynomial* $\tilde{S}\left(a_{1},a_{2},a_{3}\right)=\sigma_{2}\left(a_{1},a_{2},a_{3}\right)\cdot \sigma_{3}\left(a_{1},a_{2},a_{3}\right)=a_{1}^{2}a_{2}^{2}a_{3}+a_{1}^{2}a_{2}a_{3}^{2}+a_{1}a_{2}^{2}a_{3}^{2}$. *Then the operator*


$$\begin{array}{l} {\hat{S}}_{H}\left(\varphi \right)=\frac{1}{162}(\left(\varphi^{2}◦h_{1}\right)\cdot \left(\varphi^{2}◦h_{2}\right)\cdot \left(\varphi ◦h_{3}\right)\\ \;+\left(\varphi^{2}◦h_{1}\right)\cdot \left(\varphi ◦h_{2}\right)\cdot \left(\varphi^{2}◦h_{3}\right)\\ \;+\left(\varphi ◦h_{1}\right)\cdot \left(\varphi^{2}◦h_{2}\right)\cdot \left(\varphi^{2}◦h_{3}\right)) \end{array}$$

*is a GENEO*.

**Remark 5.5**. *It is worth noticing that if we replace the constant *C*, defined as in (5.4), with any constant* $\overset{-}{C}\geq C$, *the operator* ${\hat{S}}_{H}$ *defined as in Theorem 5.1 is still a GENEO. However, it should be specified that the larger the constant* $\overset{-}{C}$ *is, the more difficult it becomes to distinguish different signals, since their distance is smaller. For this reason, a larger* $\overset{-}{C}$ *means that more information is lost when applying the GENEO. Nonetheless, in some cases rough constants which are easier to compute may be preferred. For this reason, we present here a possible alternative to *C*. Let* $c=\max|c_{k_{1},\ldots ,k_{n}}|,m=\underset{j}{\max}m_{j},\mu =\prod_{j=1}^{n}\left(m_{j}+1\right)$ *and* ${\overset{-}{M}}_{{\text{Im}\left(\Phi \right)}^{n}}=\max\left\{M_{{\text{Im}\left(\Phi \right)}^{n}},1\right\}$, *where* $M_{{\text{Im}\left(\Phi \right)}^{n}}$ *is defined as in (5.1). We define the constant*


(5.5) C-=cμn2m(n⌈n2⌉)mnM-Im(Φ)nmn2-1.

*We can easily show that* $\overset{-}{C}\geq C$. *Therefore, the operator* ${\overset{-}{S}}_{H}$, *obtained by replacing *C* with* $\overset{-}{C}$ *in the definition of* ${\hat{S}}_{H}$ *and applying Theorem 5.1, is still a GENEO. In Example 5.2,* $\overset{-}{C}=2,304$, *which is far larger than C* = 40. *In Example 5.3*, $\overset{-}{C}=82,944$, *while C* = 1, 040. *Finally, in Example 5.4* $\overset{-}{C}=972$ *and C* = 162. *We stress that the constant *C* defined in (5.4) is optimal if we do not add any further assumption. We can realize this by applying Theorem 5.1 to the symmetric function* $\tilde{S}\left(a_{1}\right)=\sigma_{1}\left(a_{1}\right)=a_{1}$ *in just one variable, provided that* Φ *is the collection of all non-expansive functions from X*: = [0, 1] *to itself, and we set both *G* and the permutant *H* equal to the trivial group containing only the identity of *X*. On the one hand, it can be easily checked that in this case the GEO* ${\tilde{S}}_{H}$ *defined by applying Proposition 3.3 is the identity map, and hence a GENEO. Therefore, any constant *C*′ that could replace *C* in the definition of* ${\hat{S}}_{H}$ *must be not smaller than 1 in order to preserve the non-expansivity of* ${\hat{S}}_{H}$. *On the other hand, we can immediately see that* $C=M_{1}=M_{2}=M_{{\text{Im}\left(\Phi \right)}^{n}}=1$. *It follows that C*′ ≥ *C*.

## 6. GENEOs Increase Our Ability to Distinguish Data

In this section, we illustrate a few examples showing that our approach can produce useful non-linear GENEOs and increase our ability to distinguish data. As already discussed in the Introduction, it is of fundamental importance to make a large number of GENEOs available in machine learning, as each of them models a data-observer pair. The results presented in this paper could be of great help in the task of extending the set of available GENEOs and the consequent possibilities of using them in applications.

The next example shows that our new method indeed extends the approach introduced in Botteghi et al. (2020).

**Example 6.1**. Let us set *X* = {1, 2, 3} and Φ equal to the set of all functions from *X* to [0, 1]. In this case, Aut_Φ_(*X*) = *S*_3_, and we define *G* = *S*_3_. We now consider the symmetric function $\tilde{S}\left(a_{1},a_{2}\right)=a_{1}\cdot a_{2}=\sigma_{2}\left(a_{1},a_{2}\right)$ and the permutant *H* = {(1, 2, 3), (1, 3, 2)} =:{*h*_1_, *h*_2_}. From Theorem 5.1 we get that ${\hat{S}}_{H}\left(\varphi \right)=\frac{\left(\varphi ∙h_{1}\right)\cdot \left(\varphi ∙h_{2}\right)}{4}$ is a GENEO. Such an operator is not linear, and hence it cannot be obtained by the method described in Botteghi et al. (2020).

In next Example 6.2 we illustrate the synergy between GENEOs and TDA. TDA is mainly grounded on Persistent Homology (PH), which is an algebraic topological theory devised to describe “holes” in geometrical data, focusing on their persistence under the action of noise. In particular, TDA takes benefit from the topological comparison of data by means of persistence diagrams, which are the main tools in PH. For more details about TDA and PH, we refer the interested reader to Edelsbrunner and Harer (2008), Edelsbrunner and Morozov (2013). Example 6.2 shows that the use of new GENEOs increases our ability of distinguishing data by persistence diagrams.

**Figure 1:**
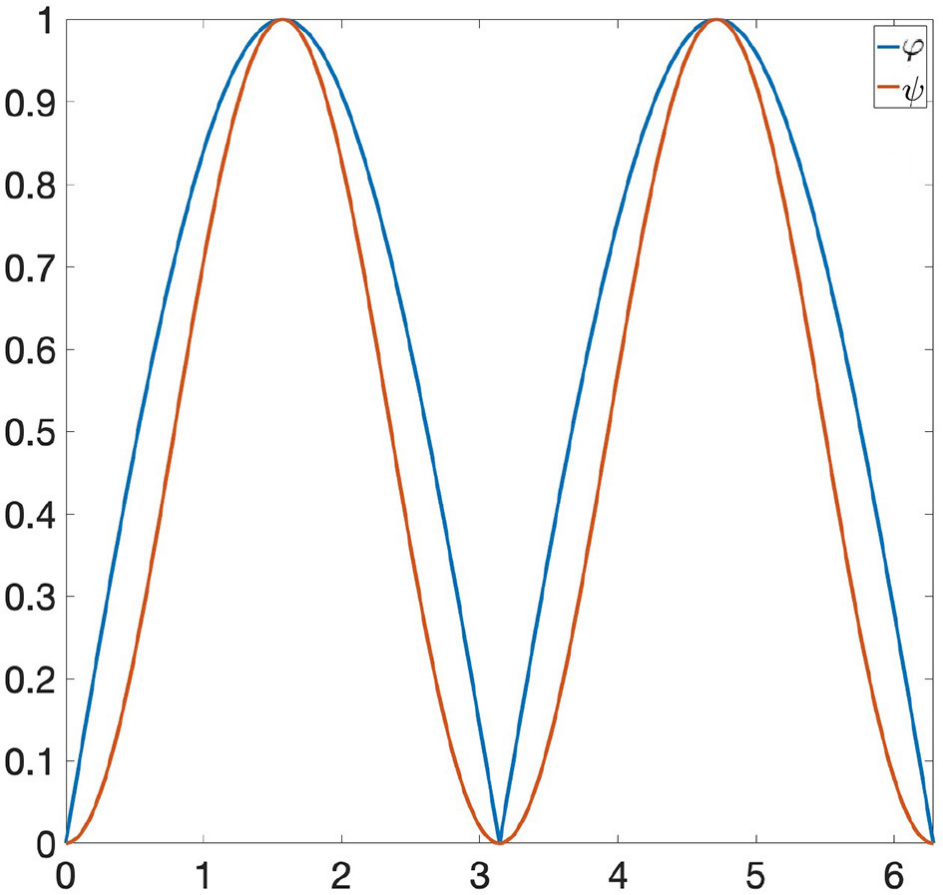
The functions φ and ψ have the same persistence diagram.

**Figure 2:**
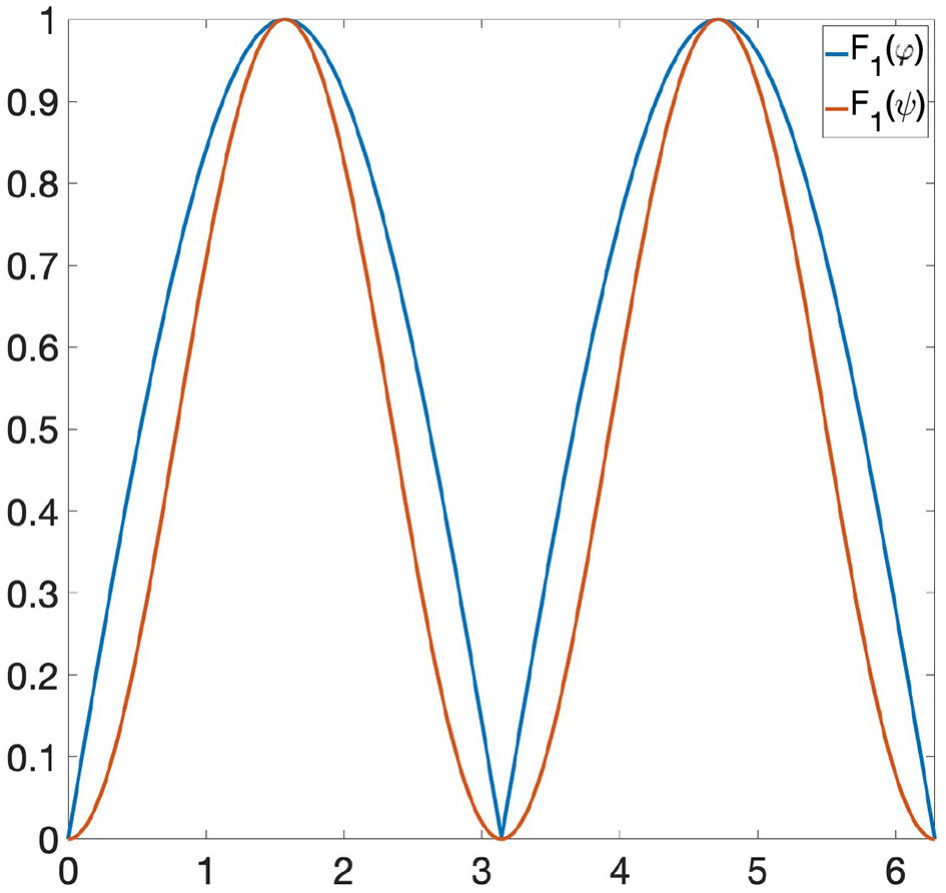
*F*_1_(φ) and *F*_1_(ψ) have the same persistence diagram.

**Figure 3:**
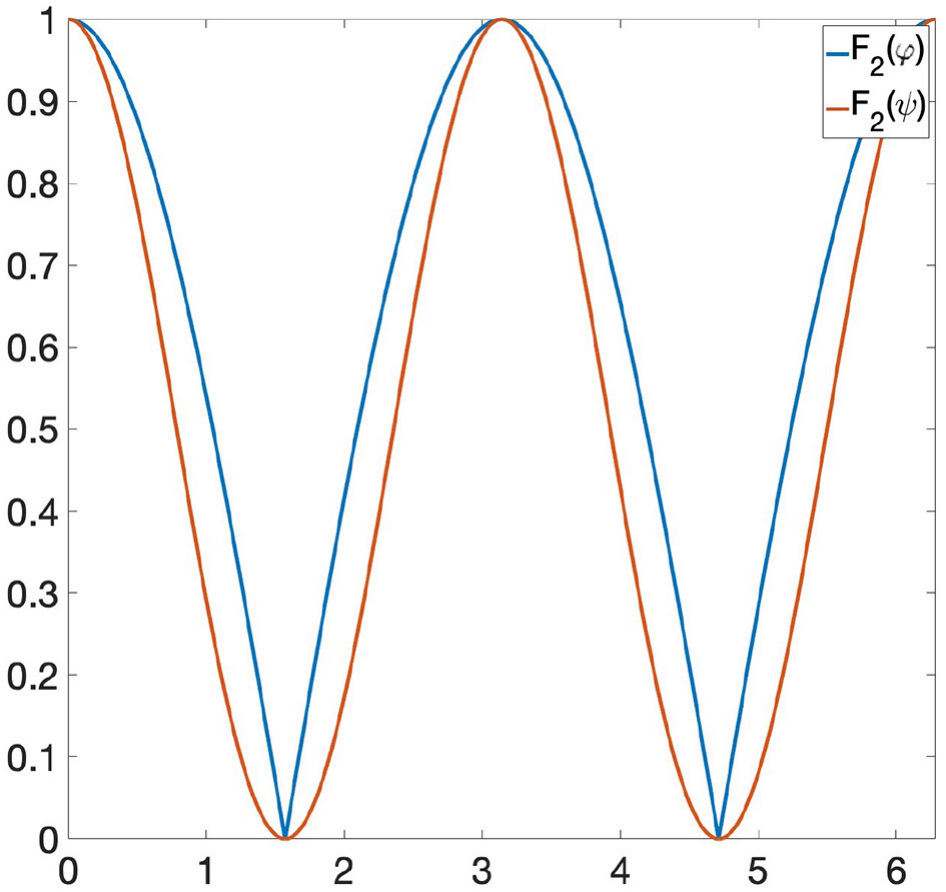
*F*_2_(φ) and *F*_2_(ψ) have the same persistence diagram.

**Figure 4:**
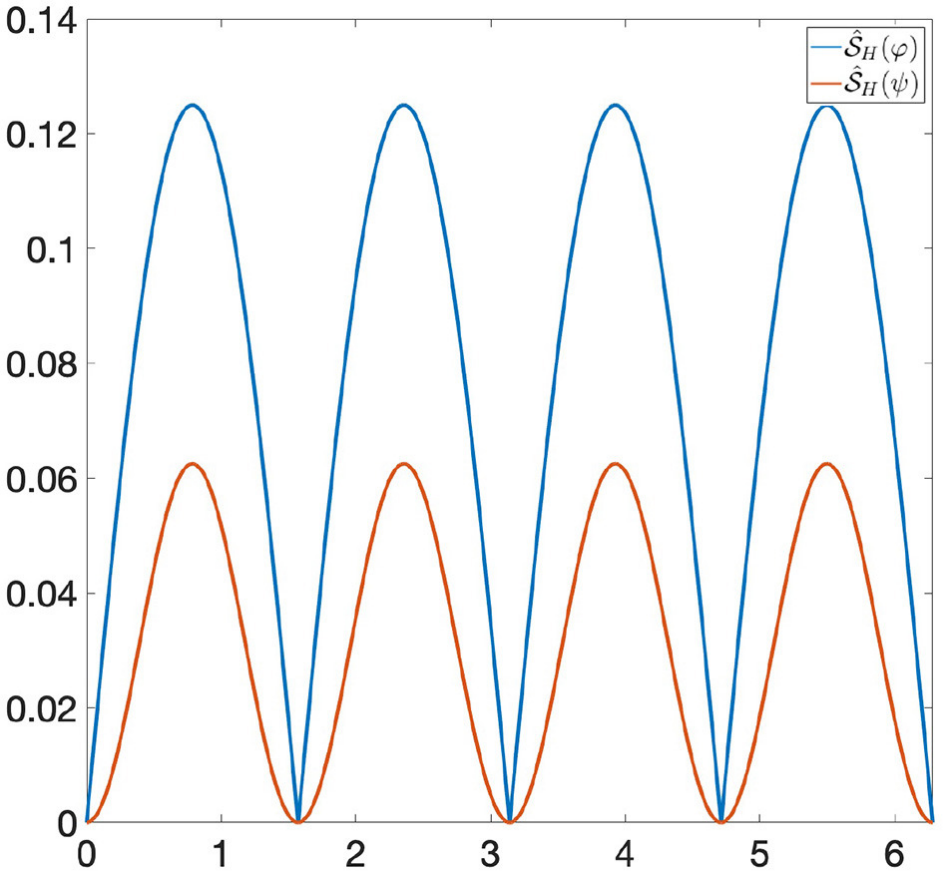
${\hat{S}}_{H}\left(\varphi \right)$ and ${\hat{S}}_{H}\left(\psi \right)$ have different persistence diagrams, and hence they are distinguishable by Persistent Homology. The bottleneck distance between their persistence diagrams is 0.0625.

**Example 6.2**. *Let us consider the following functions: φ*(*x*) = |sin(*x*)| *and ψ*(*x*) = sin(*x*)^2^ ∈ Φ, *where* Φ *is the space of all 1-Lipschitz functions from the unit circle S*^1^ to [0, 1] *and the invariance group *G* is composed of all rotations of S*^1^. *If we are looking at* Φ *only through Persistent Homology, then* φ *and ψ are indistinguishable, since they induce the same persistence diagram* (see Figure 1). *Let us now consider the GENEOs F*_1_ = id : Φ → Φ *and* $F_{2}\left(\varphi \right)=\varphi ∙\rho_{\frac{\pi }{2}}$, *where* $\rho_{\frac{\pi }{2}}$ *is the clockwise rotation through a* $\frac{\pi }{2}$ *angle. We highlight the fact that the joint use of TDA, F*_1_ *and F*_2_ *does not allow us to distinguish φ from ψ, since the functions F*_1_(*φ*) *and* *F*_1_(*ψ*) *have the same persistence diagram, and the same happens for F*_2_(*φ*) *and* *F*_2_(*ψ*) (see Figures 2, 3). *However, if we consider the symmetric function σ*_2_ *and the permutant* $H=\left\{{\text{id}}_{S^{1}},\rho_{\frac{\pi }{2}}\right\}$, *from Theorem 5.1 we get that* ${\hat{S}}_{H}\left(\varphi \right):=\frac{1}{4}\left(\varphi \cdot \left(\varphi ∙\rho_{\frac{\pi }{2}}\right)\right)$ *is a non-linear GENEO. We observe that the joint use of TDA and* ${\hat{S}}_{H}$ *allows us to distinguish φ and ψ, since the persistence diagrams of the functions* ${\hat{S}}_{H}\left(\varphi \right)$ *and* ${\hat{S}}_{H}\left(\psi \right)$ *are different from each other* (see Figure 4).

Finally, we stress another important aspect of the GENEOs. The use of GENEOs can be seen as a methodology for changing the max-norm metric that we use to compare functions in Φ. We can indeed change the distance ||φ_1_ − φ_2_||_∞_ into the pseudo-metric ||*F*(φ_1_) − *F*(φ_2_)||_∞_, where *F* is a GENEO. In Example 6.3 we show how the pseudo-metrics associated with non-linear GENEOs are much more flexible than those generated by linear operators, thus guaranteeing a wider range of applications.

**Example 6.3**. *Let us consider* Φ *as the set of all functions from the set with just two elements* *X* = {*A, B*} *to* [0, 1]. *The functions in* Φ *can be described by ordered pairs* (φ(*A*), φ(*B*)). *In this setting, the group *G* is composed of the permutations of two elements. As usual, in* Φ *we have the metric* *D*_Φ_(φ_1_, φ_2_) = ||φ_1_ − φ_2_||_∞_, *therefore the distance between the functions* (0, 0), (0, 1), (1, 0), (1, 1) *is always 1. Suppose that we need a GENEO *F* such that the pseudo-metric* ||*F*(φ_1_) − *F*(φ_2_)||_∞_ *vanishes between functions with a null component, while maintaining positive the distance between* (1, 1) *and the other three functions belonging to* Φ. *No linear GENEO can induce such a pseudo-metric, since if a linear transformation maps* (1, 0) *and* (0, 1) *to* (0, 0), *it must also map* (1, 1) *to* (0, 0). *It is worth noticing that through the GENEO associated with the elementary symmetric function σ*_2_(*a*_1_, *a*_2_) = *a*_1_ · *a*_2_ *and with the permutant H* = *G*, *we can obtain a pseudo-metric with the desired property*.

## 7. Conclusions

In our paper, we have introduced a new method to build GENEOs, grounded on the concepts of symmetric function and permutant. Our main goal is the one of building a good theory of GENEOs, making available methods to define and use these operators in machine learning. The main advantage of our approach is the fact that it requires no integration over the (possibly infinite and large) group *G*, but just the availability of a permutant and the computation of a symmetric function. Many lines of research still remain to be explored in this field. For example, the reader can observe that in section 3 the requirement that the function S is symmetric could be weakened without losing the property that $S_{H}$ is a GEO. It would be indeed sufficient to assume that S is invariant when we apply to its argument any permutation corresponding to the permutation of *H* associated with the conjugation action *h* ↦ *g* ◦ *h* ◦ *g*^−1^ that is defined by any *g* ∈ *G*. We have decided to postpone the research concerning this extension of the theory, since it would have added a dependence on *H* of the choice of S, thereby introducing some technicalities. Another line of research that we would like to explore in the future is the possibility of adapting our approach to permutant measures, which are a sort of extension of the concept of permutant to the case that *H* has infinite cardinality. However, the most challenging problem we will have to face is likely to be the proof or disproof of the natural conjecture that each non-linear *G*-equivariant non-expansive operator can be produced (or at least well approximated) by applying our new technique to suitable symmetric functions and permutants, provided that the group *G* transitively acts on a finite signal domain. We plan to devote some subsequent papers to these topics.

## Data Availability Statement

The original contributions presented in the study are included in the article/Supplementary Material, further inquiries can be directed to the corresponding author/s.

## Author Contributions

PF devised the project. All authors contributed to the manuscript. All authors read and approved the final manuscript.

## Conflict of Interest

The authors declare that the research was conducted in the absence of any commercial or financial relationships that could be construed as a potential conflict of interest.

## Publisher's Note

All claims expressed in this article are solely those of the authors and do not necessarily represent those of their affiliated organizations, or those of the publisher, the editors and the reviewers. Any product that may be evaluated in this article, or claim that may be made by its manufacturer, is not guaranteed or endorsed by the publisher.
