# Supplementary material for: On the Construction of Group Equivariant Non-Expansive Operators via Permutants and Symmetric Functions
Source: Front Artif Intell. 2022 Feb 15;5:786091. doi: 10.3389/frai.2022.786091 (PMC8887714; doi:10.3389/frai.2022.786091)
Supplement: Supplementary file 1 [file Presentation_1.pdf]

Appendix of the paper “On the construction of Group Equivariant Non-Expansive operators via permutants and symmetric functions”

Francesco Conti, Patrizio Frosini and Nicola Quercioli

1 APPENDIX

In this appendix we introduce some lemmas that will be of use in our paper. For any $\alpha = (\alpha_1, \ldots, \alpha_n) \in \mathbb{R}^n$, we will set $\|(\alpha_1, \ldots, \alpha_n)\|_\infty := \max_{1 \le i \le n} |\alpha_i|$.

LEMMA 1.1. *Consider $\alpha = (\alpha_1, \ldots, \alpha_n)$ and $\beta = (\beta_1, \ldots, \beta_n)$ in $\mathbb{R}^n$, for some positive integer n, and assume that at least one of these two points is different from $\mathbf{0}$. Then, for every index k with $1 \le k \le n$,*

$$(P_k) \quad \left| \prod_{i=1}^{k} \alpha_i - \prod_{i=1}^{k} \beta_i \right| \le k \|\alpha - \beta\|_\infty \max\{\|\alpha\|_\infty, \|\beta\|_\infty\}^{k-1}.$$

PROOF. We will prove the statement by induction on k. The statement is trivially true for $k = 1$. Let us now assume that the inequality (P_{k-1}) holds, with $1 \le k - 1 \le n - 1$. We have that

$$\begin{aligned}
\left| \prod_{i=1}^{k} \alpha_i - \prod_{i=1}^{k} \beta_i \right| &= \left| \alpha_k \prod_{i=1}^{k-1} \alpha_i - \beta_k \prod_{i=1}^{k-1} \beta_i \right| \\
&= \left| \alpha_k \prod_{i=1}^{k-1} \alpha_i - \alpha_k \prod_{i=1}^{k-1} \beta_i + \alpha_k \prod_{i=1}^{k-1} \beta_i - \beta_k \prod_{i=1}^{k-1} \beta_i \right| \\
&\le |\alpha_k| \left| \prod_{i=1}^{k-1} \alpha_i - \prod_{i=1}^{k-1} \beta_i \right| + |\alpha_k - \beta_k| \left| \prod_{i=1}^{k-1} \beta_i \right| \\
&\le \|\alpha\|_\infty (k-1) \|\alpha - \beta\|_\infty \max\{\|\alpha\|_\infty, \|\beta\|_\infty\}^{k-2} + \|\alpha - \beta\|_\infty \|\beta\|_\infty^{k-1} \\
&= \|\alpha - \beta\|_\infty \left(\|\alpha\|_\infty (k-1) \max\{\|\alpha\|_\infty, \|\beta\|_\infty\}^{k-2} + \|\beta\|_\infty^{k-1} \right) \\
&\le \|\alpha - \beta\|_\infty \left((k-1) \max\{\|\alpha\|_\infty, \|\beta\|_\infty\}^{k-1} + \max\{\|\alpha\|_\infty, \|\beta\|_\infty\}^{k-1} \right) \\
&= k \|\alpha - \beta\|_\infty \max\{\|\alpha\|_\infty, \|\beta\|_\infty\}^{k-1}.
\end{aligned}$$

Hence, the statement (P_k) is true for every index k with $1 \le k \le n$.

Corollary 1.2. If $a, b \in \mathbb{R}$, $a^2 + b^2 \neq 0$ and $n \in \mathbb{N} \setminus \{0\}$, then $|a^n - b^n| \le n |a - b| \cdot \max\{|a|, |b|\}^{n-1}$.

PROOF. It follows by applying Lemma 1.1 to $\alpha = (a, \ldots, a)$, $\beta = (b, \ldots, b) \in \mathbb{R}^n$, with $k = n$.

In the following, we will consider a compact subset K of $\mathbb{R}^n$, and set $M_K := \max_{\alpha \in K} \|\alpha\|_\infty$. The symbol σ_i will denote the i-th elementary symmetric polynomial, for $i \in \{1, \ldots, n\}$ (Definition 4.5).

LEMMA 1.3. *For every $\alpha \in K$ and $i \in \{1, \ldots, n\}$, the inequality $|\sigma_i(\alpha)| \le \binom{n}{i} M_K^i$ holds.*

PROOF. For every $\alpha = (\alpha_1, \dots, \alpha_n) \in K$, we have that

$$\begin{aligned}
|\sigma_i(\alpha)| &= \left| \sum_{1 \le j_1 < \dots < j_i \le n} \alpha_{j_1} \cdot \ldots \cdot \alpha_{j_i} \right| \\
&\le \sum_{1 \le j_1 < \dots < j_i \le n} |\alpha_{j_1} \cdot \ldots \cdot \alpha_{j_i}| \\
&\le \binom{n}{i} \|\alpha\|_\infty^i \\
&\le \binom{n}{i} M_K^i.
\end{aligned}$$

LEMMA 1.4. *For every $\alpha, \beta \in K$ and $i \in \{1, \dots, n\}$, $|\sigma_i(\alpha) - \sigma_i(\beta)| \le \binom{n}{i} i \|\alpha - \beta\|_\infty M_K^{i-1}$.*

PROOF. The statement is trivial for $\alpha = \beta$, hence we will consider the case $\alpha \ne \beta$. Assume $\alpha = (\alpha_1, \dots, \alpha_n), \beta = (\beta_1, \dots, \beta_n) \in \mathbb{R}^n$. By Lemma 1.1, we get

$$\begin{aligned}
|\sigma_i(\alpha) - \sigma_i(\beta)| &= \left| \sum_{1 \le j_1 < \dots < j_i \le n} \alpha_{j_1} \cdot \ldots \cdot \alpha_{j_i} - \sum_{1 \le j_1 < \dots < j_i \le n} \beta_{j_1} \cdot \ldots \cdot \beta_{j_i} \right| \\
&\le \sum_{1 \le j_1 < \dots < j_i \le n} |\alpha_{j_1} \cdot \ldots \cdot \alpha_{j_i} - \beta_{j_1} \cdot \ldots \cdot \beta_{j_i}| \\
&\le \sum_{1 \le j_1 < \dots < j_i \le n} i \|\alpha - \beta\|_\infty \max \left\{ \|\alpha\|_\infty, \|\beta\|_\infty \right\}^{i-1} \\
&= \binom{n}{i} i \|\alpha - \beta\|_\infty \max \left\{ \|\alpha\|_\infty, \|\beta\|_\infty \right\}^{i-1} \\
&\le \binom{n}{i} i \|\alpha - \beta\|_\infty M_K^{i-1}.
\end{aligned}$$

LEMMA 1.5. *For every $\alpha, \beta \in K$, every positive integer n, and every $(k_1, \dots, k_n) \in \mathbb{N}^n$*

$$\left| \prod_{i=1}^n \sigma_i^{k_i}(\alpha) - \prod_{i=1}^n \sigma_i^{k_i}(\beta) \right| \le n \|\alpha - \beta\|_\infty \max_{1 \le i \le n} \left\{ k_i \binom{n}{i}^{k_i} i M_K^{ik_i - 1} \right\} \max_{1 \le i \le n} \left\{ \binom{n}{i}^{k_i} M_K^{ik_i} \right\}^{n-1}.$$

PROOF. Let us set $\sigma^k(\alpha) := \left(\sigma_1^{k_1}(\alpha), \dots, \sigma_n^{k_n}(\alpha) \right)$ and $\sigma^k(\beta) := \left(\sigma_1^{k_1}(\beta), \dots, \sigma_n^{k_n}(\beta) \right)$. From Lemma 1.1 we get the following inequality:

$$\left| \prod_{i=1}^n \sigma_i^{k_i}(\alpha) - \prod_{i=1}^n \sigma_i^{k_i}(\beta) \right| \le n \left\| \sigma^k(\alpha) - \sigma^k(\beta) \right\|_\infty \max \left\{ \left\| \sigma^k(\alpha) \right\|_\infty, \left\| \sigma^k(\beta) \right\|_\infty \right\}^{n-1}.$$

From Lemma 1.3 we get that:

$$\left\| \sigma^k(\alpha) \right\|_\infty = \max_{1 \le i \le n} \left\{ \left| \sigma_i^{k_i}(\alpha) \right| \right\} \le \max_{1 \le i \le n} \left\{ \binom{n}{i}^{k_i} M_K^{ik_i} \right\}.$$

Analogously, $\left\|\sigma^k(\beta)\right\|_\infty \leq \max_{1\leq i\leq n}\left\{\binom{n}{i}^{k_i} M_K^{ik_i}\right\}$. Therefore:

$$\max\left\{\left\|\sigma^k(\alpha)\right\|_\infty, \left\|\sigma^k(\beta)\right\|_\infty\right\}^{n-1} \leq \max_{1\leq i\leq n}\left\{\binom{n}{i}^{k_i} M_K^{ik_i}\right\}^{n-1}.$$

From Corollary 1.2, Lemma 1.3 and Lemma 1.4 we get that:

$$\begin{aligned}
\left\|\sigma^k(\alpha)-\sigma^k(\beta)\right\|_\infty &= \max_{1\leq i\leq n}\left\{\left|\sigma_i^{k_i}(\alpha)-\sigma_i^{k_i}(\beta)\right|\right\} \\
&\leq \max_{1\leq i\leq n}\left\{k_i\left|\sigma_i(\alpha)-\sigma_i(\beta)\right|\max\left\{\left|\sigma_i(\alpha)\right|,\left|\sigma_i(\beta)\right|\right\}^{k_i-1}\right\} \\
&\leq \max_{1\leq i\leq n}\left\{k_i\binom{n}{i} i\left\|\alpha-\beta\right\|_\infty M_K^{i-1}\binom{n}{i}^{k_i-1} M_K^{i(k_i-1)}\right\} \\
&= \left\|\alpha-\beta\right\|_\infty \max_{1\leq i\leq n}\left\{k_i\binom{n}{i}^{k_i} iM_K^{ik_i-1}\right\}.
\end{aligned}$$

In conclusion,

$$\left|\prod_{i=1}^n \sigma_i^{k_i}(\alpha)-\prod_{i=1}^n \sigma_i^{k_i}(\beta)\right| \leq n\left\|\alpha-\beta\right\|_\infty \max_{1\leq i\leq n}\left\{k_i\binom{n}{i}^{k_i} iM_K^{ik_i-1}\right\}\max_{1\leq i\leq n}\left\{\binom{n}{i}^{k_i} M_K^{ik_i}\right\}^{n-1}.$$
